# The clinical relevance of neutrophil-to-lymphocyte ratio and platelet-to-lymphocyte ratio in chronic obstructive pulmonary disease with lung cancer

**DOI:** 10.3389/fonc.2022.902955

**Published:** 2022-09-27

**Authors:** Aiping Ma, Guangdong Wang, Yan Du, Weixi Guo, Jiaxi Guo, Yi Hu, Dongyu Bai, Huiping Huang, Lianjin Zhuang, Jinhan Chen, Qun Liu

**Affiliations:** ^1^Department of Respiratory and Critical Medicine, The First Affiliated Hospital, School of Medicine, Xiamen University, Xiamen, China; ^2^Department of Thoracic Surgery, The First Affiliated Hospital, School of Medicine, Xiamen University, Xiamen, China; ^3^Department of Clinical Laboratory, The First Affiliated Hospital, School of Medicine, Xiamen University, Xiamen, China; ^4^Department of Pathology, The First Affiliated Hospital, School of Medicine, Xiamen University, Xiamen, China; ^5^Department of Infection Control, The First Affiliated Hospital, School of Medicine, Xiamen University, Xiamen, China; ^6^Division of Quality Management, The First Affiliated Hospital, School of Medicine, Xiamen University, Xiamen, China; ^7^Department of Geriatrics, The First Affiliated Hospital, School of Medicine, Xiamen University, Xiamen, China

**Keywords:** chronic obstructive pulmonary disease, lung cancer, neutrophil-to-lymphocyte ratio, platelet-to-lymphocyte ratio, inflammation biomarkers

## Abstract

**Background:**

Chronic obstructive pulmonary disease (COPD) coexisting with lung cancer is associated with severe mortality and a worse prognosis. Inflammation plays an important role in common pathogenic pathways and disease progression. However, a few studies have identified the clinical value of the neutrophil-to-lymphocyte ratio (NLR) and platelet-to-lymphocyte ratio (PLR) in COPD with lung cancer, which are systemic inflammatory response markers in the blood. This study aimed to determine the association of the NLR or PLR with clinical characteristics and whether NLR or PLR can be diagnostic markers for COPD with lung cancer.

**Methods:**

Between 2015 and 2021, we conducted a retrospective analysis of 236 COPD patients with lung cancer and 500 patients without lung cancer (control group). Clinical information, blood routine examination, and spirometry results were collected and analyzed. The receiver operating characteristic (ROC) curve was used to identify the best cutoff point of NLR or PLR. Multivariate logistic regression analysis was performed to evaluate the association of NLR or PLR with the diagnosis and prognosis of COPD with lung cancer.

**Results:**

Compared to patients in the COPD-only group, patients in the lung cancer group had a higher percentage of current smoking and emphysema, and it was found that NLR or PLR was significantly higher in the lung cancer group. Multivariate analysis showed that age, smoking status, FEV1%pred, emphysema, NLR, and PLR were independent risk factors for lung cancer development in COPD. Furthermore, the high level of NLR or PLR was associated with age over 70 years old, current smoking status, and ineligible surgery treatment. The level of PLR or NLR markedly increased with hypercoagulation status, the severity of airflow limitation, and advanced progression of lung cancer. Additionally, the ROC analysis also revealed that elevated NLR or PLR was an independent predictor of COPD in lung cancer patients, TNM stages IIIB–IV at first diagnosis in lung cancer, and ineligible surgery in lung cancer patients.

**Conclusion:**

Increased NLR or PLR values might be an important and easily measurable inflammation biomarker to predict the diagnosis and severity of lung cancer with COPD.

## Introduction

Chronic obstructive pulmonary disease (COPD) and lung cancer comprise the major causes of lung disease-related deaths worldwide (1). It was estimated that the total number of newly diagnosed cases of lung cancer in China in 2015 was about 787,000 (2). Furthermore, COPD is one of the leading causes of morbidity and mortality worldwide (3). In 2016, COPD was the fifth leading cause of death in China (4). Overall, lung cancer and COPD present a major public health issue and an enormous burden on society in China (5–7). Several studies describe that there is an association between COPD and the development of lung cancer. An increase in the incidence and mortality of lung cancer was observed in patients with COPD (8). Machida and colleagues investigated the incidence of lung cancer in the stable COPD population which was 1.85% per year during a median follow-up period of 4.58 years (9). Moreover, the prevalence of COPD in lung cancer patients was 32.6%, and with the increased severity of COPD, the prognosis of lung cancer gradually worsened (10). Current research has indicated that lung cancer accounts for 12% to 14% of COPD-related deaths (11, 12). Additionally, never-smokers of non-small cell lung cancer (NSCLC) patients with COPD had shorter overall survival (OS) times, compared to non-COPD never-smoker NSCLC patients (13). It has been also found that even in patients with early-stage COPD, the prevalence of postoperative pulmonary complications (PPCs) was higher than in patients with NSCLC with normal spirometry (14). Therefore, patients with lung cancer and coexisting COPD may have a much worse prognosis than those with COPD only.

The common pathogenic pathways contributing to both diseases share underlying etiologies, such as tobacco, gene expressions, susceptibility to DNA damage, environmental factors, and inflammation (1, 15, 16). However, more details of the relationship between COPD and lung cancer remain uncertain. Some studies have demonstrated that chronic inflammation could be one of the underlying mechanisms, which has been associated with carcinogenesis in lung cancer, airway remodeling, and severe comorbidities in COPD (17–20). Although some studies have demonstrated that high levels of several inflammation markers were related to progression-free survival (PFS) and OS in lung cancer patients with and without COPD, there is still a lack of effective markers to evaluate COPD with lung cancer (21).

The neutrophil-to-lymphocyte ratio (NLR) and platelet-to-lymphocyte ratio (PLR) are the available markers of systemic inflammation and routine clinical laboratory tests (22–24). Recent studies have indicated that NLR and PLR were higher in acute exacerbation of COPD and used for predicting hospitalization (3, 25). Furthermore, they were related to short-term mortality in patients hospitalized with acute exacerbation of COPD (AECOPD) (26, 27). NLR and PLR values were also found to be higher in advanced lung cancer (28). It was reported that NLR has been associated with worse survival and recurrence (29). The combination of NLR and PLR has increased the diagnostic value and distinguished lung cancer patients from healthy subjects (30). However, few studies have reported whether NLR and PLR could better evaluate COPD with lung cancer. In the study, we aimed to identify the baseline clinical characteristics of COPD coexisting with lung cancer and investigate whether NLR or PLR can be diagnostic markers for COPD with lung cancer.

## Materials and methods

### Study population

We retrospectively reviewed the medical records of 1,264 patients diagnosed with COPD between January 2015 and August 2021 at the inpatient clinic of our respiratory department at The First Affiliated Hospital of Xiamen University, Fujian, People’s Republic of China. Patients who have incomplete medical records, with contraindications, or are unable to perform spirometry correctly and those who were diagnosed with asthma and interstitial lung disease were excluded. Finally, a total of 736 patients were eligible for this study. All participants underwent spirometry to measure pulmonary function, and their complete medical records were retrieved for analysis. Spirometry was done according to the American Thoracic Society recommendations with central quality assurance of spirometry tracings (19). Among the 1,264 patients, a total of 236 COPD patients with lung cancer were diagnosed through histological and/or cytological specimens.

### Data collection

Basic clinical information on age, sex, smoking status, body mass index (BMI), lung cancer diagnosis, histologic type, routine blood, coagulation, and cancer stage was taken from the medical records for all patients. As a retrospective study, all data were anonymous. We declared that the patients’ data were confidential and did not compromise the patients’ interests. Data collection was completed by two independent authors. The ethics approval of the study was obtained from the Clinical Research Ethics Committee of the First Affiliated Hospital of Xiamen University (2021064).

### Lung cancer group

Histologic lung cancer was categorized as small-cell lung cancer (SCLC) and non-SCLC (NSCLC) according to the 2015 WHO classification of lung tumors (31). NSCLC was then further categorized as adenocarcinoma, squamous cell carcinoma (SCC), or other, which consisted primarily of large-cell carcinoma and undifferentiated carcinoma of the lungs. Cancer was staged in accordance with the Union for International Cancer Control, Tumour, Node, Metastasis 7 (TNM7) classification and staging I–IV. All lung cancer patients with COPD were diagnosed through CT-guided lung tissue biopsy, thoracic surgery, or bronchoscopic biopsy.

### Routine blood

Peripheral venous blood was collected from all patients into Vacutainer tubes in the morning on the first day of admission and processed immediately. The differential blood counts (white blood cell, neutrophil, lymphocyte) and platelet count were measured using the hematology analyzer Sysmex XN-9000 (XN, Sysmex, Kobe, Japan).

### Assessment of COPD

Spirometry tests were performed by qualified technicians on a computerized spirometer MasterScreen, (Jaeger, Freistaat Bayern, Germany), following the criteria for the standardization of pulmonary function tests recommended by the American Thoracic Society/European Respiratory Society (ATS/ERS) Task Force (32). According to The Global Initiative for Chronic Obstructive Lung Disease (GOLD) (http://goldcopd.org) guidelines, forced vital capacity (FVC) and forced expiratory volume in 1 s (FEV1) were recorded in medical records in liters and percentage predicted values, as well as FEV1/FVC%. The post-bronchodilator fixed criteria FEV1/FVC <0.7 was applied to define COPD. The GOLD criteria were also used to assign a grade of clinical severity to COPD based on FEV1%pred and FEV1/FVC: patients with an FEV1/FVC <0.7 were classified as having COPD in all grades; GOLD 1 was defined as having an FEV1%pred >80; GOLD 2 as 50 ≤ FEV1%pred <80; GOLD 3 as 30% ≤ FEV1%pred <50%; and GOLD 4 as FEV1%pred <30% (33).

### Statistical analysis

All patient data were presented in terms of frequencies and mean ± standard deviations as appropriate. Normally distributed continuous variables were evaluated using the unpaired Student’s *t*-test. Continuous variables with a skewed distribution were analyzed using the Mann–Whitney *U* test. Categorical variables were assessed using the chi-square test. Correlations were done using Pearson’s correlation, and “*r*” is the correlation coefficient. It ranged from −1 to +1. Receiver operating characteristic (ROC) curve analysis was used to evaluate the sensitivity and specificity of NLR and PLR for predicting the diagnosis of COPD with lung cancer. We performed a univariate analysis with age, BMI, smoking status, FEV1%pred, NLR, PLR, and emphysema. The variables with *P <*0.05 in the univariate analysis were entered into a multivariate logistic regression analysis. *P*-value <0.05 was assumed for statistical significance in all the tests. Data were analyzed with SPSS 26.0 (SPSS Inc., Chicago, IL, USA).

## Results

### General characteristics of the patients

**Figure 1:**
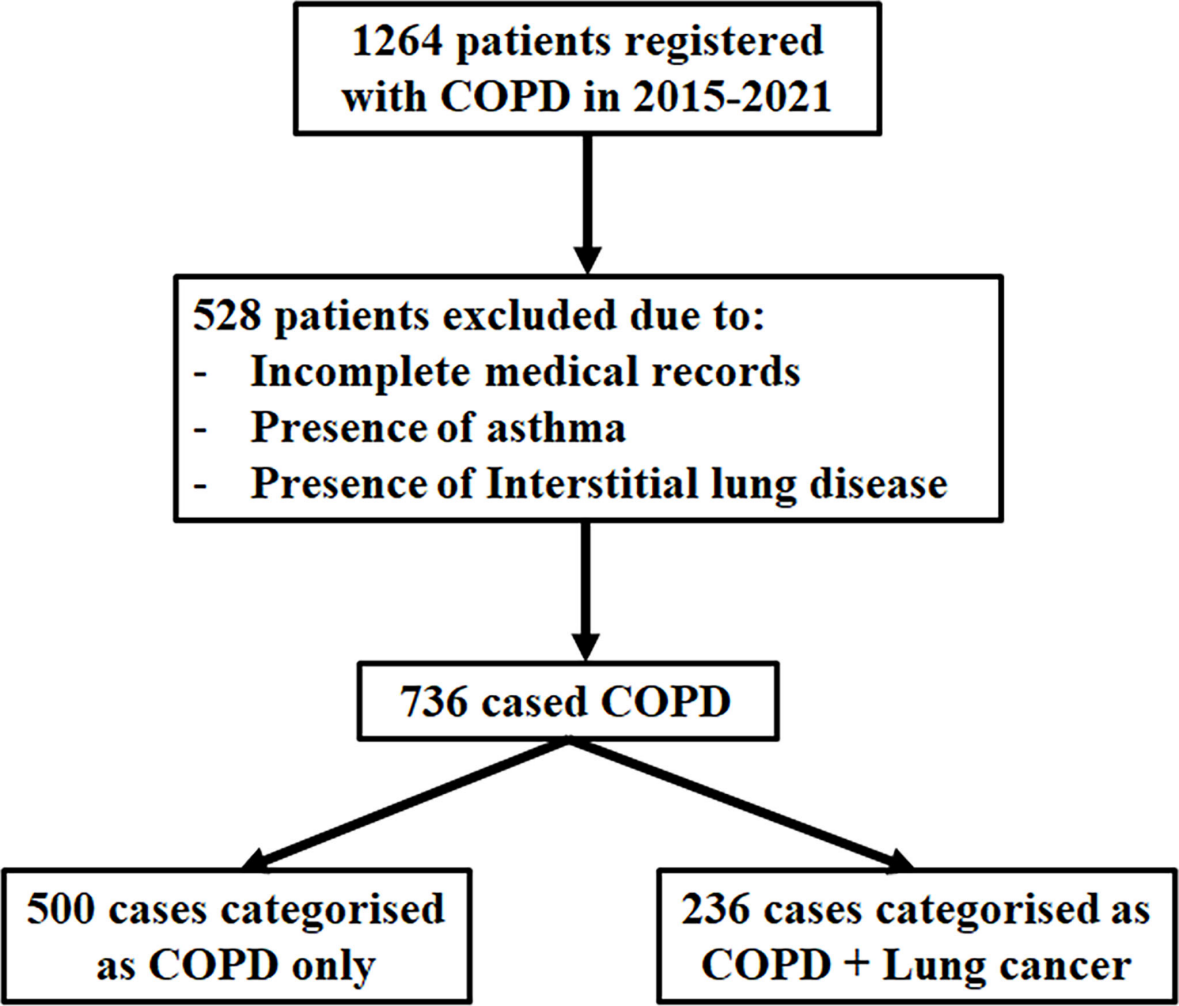
Flowchart of the population included in the study. Between 2015 and 2021, a total of 1,264 patients were retrospectively reviewed; 528 were excluded from the analysis due to missing medical records, asthma, and interstitial lung disease. The number of patients with COPD (FEV1/FVC < 70%) was 736, which were eligible for this study. Of the 736 patients included, 236 cases had COPD with lung cancer, and 500 controls had COPD only.

A total of 736 patients were eligible for this study: 236 patients were diagnosed as having COPD with lung cancer, and 500 patients were categorized as COPD (**Figure 1**). Patients’ baseline characteristics included median age, gender, BMI, smoking history, pulmonary function, and inflammatory markers (**Table 1**). As shown in the table, there were no significant differences between BMI and gender. The median onset age was earlier in the COPD with lung cancer group than in the COPD group (66.44 ± 8.37 vs. 69.87 ± 9.37, *P* < 0.001). The proportion of current smoking was significantly higher in the COPD with lung cancer group [178 (75.4%)] than that in the COPD-only group [254 (50.8%)]. Moreover, the proportion of ever smoker was significantly lower in the COPD with lung cancer group [58 (24.6%)] compared with that in the COPD-only group [246 (49.2%)] (*P* < 0.001). In addition, there were significant differences in the degree of airflow obstruction in lung function. The values for FEV1/FVC and FEV1% predicted were all significantly higher in the COPD with lung cancer group than in the COPD group (0.59 ± 0.10 vs. 0.52 ± 0.11, *P* < 0.001; 66.46 ± 20.54 vs. 47.39 ± 21.01, *P* < 0.001, respectively).

**Table 1:** Baseline characteristics of the study population.

Clinical characteristics	COPD with lung cancer (*n* = 236)	COPD only (*n* = 500)	*P*-value
**Age (years)**	66.44 ± 8.37	69.87 ± 9.37	<0.001
**Gender, *n* (%)**			
Male	223 (94.5)	470 (94.0)	0.791
Female	13 (5.5)	30 (6.0)	
**BMI (kg/m^2^)**	21.18 ± 2.58	20.79 ± 3.29	0.114
**Smoking status, *n* (%)**			
Current smoking	178 (75.4)	254 (50.8)	<0.001
Never/ever smoking	58 (24.6)	246 (49.2)	
**Pulmonary functions**			
FEV1/FVC	0.59 ± 0.10	0.52 ± 0.11	<0.001
FEV1%pred	66.46 ± 20.54	47.39 ± 21.01	<0.001
**Inflammatory markers**			
WBC (10^9^/L)	8.68 ± 1.60	6.69 ± 1.60	<0.001
Neutrophil (10^9^/L)	6.03 ± 1.29	4.13 ± 1.24	<0.001
Lymphocyte (10^9^/L)	1.75 ± 0.37	1.78 ± 0.51	0.641
NLR	3.51 ± 0.72	2.45 ± 0.81	<0.001
PLR	172.72 ± 24.96	142.64 ± 37.56	<0.001
**Emphysema, *n* (%)**			
Yes	205 (86.9)	341 (68.2)	<0.001
No	31 (13.1)	159 (31.8)	

BMI, body mass index; FEV1, forced expiratory volume in 1 s; FVC, forced vital capacity; WBC, white blood cell; NLR, neutrophil-to-lymphocyte ratio; PLR, platelet-to-lymphocyte ratio.

Next, to verify the predictive value of inflammatory markers in COPD with lung cancer, peripheral blood analyses of total white blood cell, neutrophil counts, lymphocyte, NLR, and PLR were measured. The results indicated that COPD with lung cancer patients had significantly higher levels of white blood cell and neutrophil counts (*P* < 0.001). Moreover, a statistical significance was found in NLR or PLR between COPD and COPD coexisting with lung cancer. The levels of NLR and PLR were significantly increased in the COPD with lung cancer group compared with those in the COPD group (3.51 ± 0.72 vs. 2.45 ± 0.81, 172.72 ± 24.96 vs. 142.64 ± 37.56, *P* < 0.001). Finally, the results showed a significant increase in the proportion of emphysema in the COPD with lung cancer group than in the COPD group.

### Baseline characteristics of COPD patients with lung cancer

Of the total population, 236 cases were diagnosed with lung cancer. Histologic type analyses showed that adenocarcinoma was the most frequent histological subtype in the COPD with lung cancer group (51.3%), and 30.9% were diagnosed with squamous cell carcinoma, 3.4% with large cell lung cancer, and 13.1% with SCC (**Table 2**). Among the 236 patients, 132 (55.9%) had lung cancer located in the right lobe of the lungs. Moreover, the TNM stage of lung cancer in COPD patients tended to be advanced, with stages III–VI accounting for 63.6% (**Supplementary Table 1**).

**Table 2:** Multivariate analysis of independent predictors of (A) lung cancer diagnosis in COPD patients, (B) TNM stages IIIB–IV at first diagnosis in the COPD with lung cancer patients, and (C) ineligible surgery at first diagnosis in the COPD with lung cancer patients.

Risk factors	OR	95% CI	*P*-value
**A**			
**Age (≥70)**	0.291	0.179–0.474	<0.001
**Smoking status**			
Current smoking	1.868	1.143–3.053	0.013
Never/ever smoking	–	–	–
**FEV1%pred**	1.049	1.038–1.061	<0.001
**NLR**			
NLR ≥ 2.91	12.731	7.493–21.631	<0.001
NLR < 2.91	–	–	–
**PLR**			
PLR ≥ 156.53	4.389	2.609–7.382	<0.001
PLR < 156.53	–	–	–
**Emphysema**	2.815	1.560–5.080	0.001
**B**			
**Smoking status**			
Current smoking	6.583	2.941–14.736	<0.001
Never/ever smoking	–	–	–
**NLR**			
NLR ≥ 3.53	3.788	1.811–7.923	<0.001
NLR < 3.53	–	–	–
**PLR**			
PLR ≥ 172.10	4.775	2.321–9.823	<0.001
PLR < 172.10	–	–	–
**C**			
**BMI**			
≥25 (kg/m^2^)	0.219	0.061–0.792	0.021
<25(kg/m^2^)	–	–	–
**Smoking status**			
Current smoking	4.267	2.051–8.875	<0.001
Never/ever smoking	–	–	–
**PLR**			
PLR ≥ 172.10	3.268	1.531–6.976	0.002
PLR < 172.10	–	–	–

BMI, body mass index; FEV1, forced expiratory volume in 1 s; NLR, neutrophil-to-lymphocyte ratio; PLR, platelet-to-lymphocyte ratio.

### The levels of NLR or PLR in COPD with lung cancer

In order to confirm whether NLR or PLR served as an independent inflammation biomarker for predicting the progression of lung cancer in COPD patients, the level of NLR or PLR was compared among these groups with different clinical features. The results showed that there were no significant differences in NLR or PLR values between gender and BMI (**Supplementary Table 2**). NLR or PLR levels could markedly increase with advanced age (*P* < 0.001). In addition, the level of NLR or PLR in current smoker was higher than in never/ever smoker (*P* < 0.05), and the NLR or PLR was significantly higher in GOLD stages 3–4 than 1–2 (*P* < 0.001). Moreover, lung cancer patients at TNM stages IIIB–IV had higher levels of NLR and PLR than those at stages I–IIIA (*P* < 0.001). Finally, the ineligible surgery group had higher levels of NLR and PLR than the eligible for surgery lung cancer patients (*P* < 0.001).

### The correlation between NLR or PLR with clinical characteristics in COPD with lung cancer

To further evaluate the correlation between NLR or PLR and other clinical parameters, Pearson’s correlation coefficients were analyzed. It was indicated that a positive correlation was observed between NLR or PLR value and age, PT, FIB, and D-dimer (*P* < 0.05) (**Supplementary Figure 1**). Conversely, NLR or PLR value was negatively correlated with FEV1%pred (*r* = −0.299, *P* < 0.001; *r* = −0.300, *P* < 0.001, respectively). We also found a negative correlation between PLR and BMI (*r* = −0.146, *P* = 0.025). In addition, PLR was positively correlated with PLT and APTT (*r* = 0.167, *P* < 0.01; *r* = 0.148, *P* = 0.023, respectively).

### Predictive role of NLR or PLR in COPD with lung cancer

**Figure 2:**
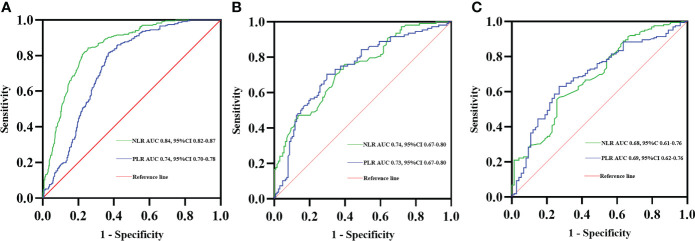
Receiver operating characteristic (ROC) curves of NLR and PLR predicting lung cancer diagnosis in COPD patients **(A)**. ROC curves of NLR and PLR predicting TNM stages IIIB–IV at first diagnosis in the COPD with lung cancer patients **(B)**. ROC curves of NLR and PLR predicting ineligible surgery at first diagnosis in the COPD with lung cancer patients **(C)**.

ROC curve analysis was also performed for the NLR and PLR values to detect the diagnosis of lung cancer in COPD patients. The best NLR cutoff value was defined as 2.91, the sensitivity was 84.3%, and the specificity was 74.4%, with the best AUC of 0.84. The optimal PLR cutoff value was 156.53 and the AUC was 0.74, with 81.8% sensitivity and 62.8% specificity (**Figure 2A**). Moreover, the ROC curve analysis using NLR and PLR to predict TNM stages IIIB–IV at first diagnosis in the COPD with lung cancer patients indicated an optimal cutoff NLR of 3.53, AUC of 0.74, sensitivity of 60.9%, and specificity of 75.9%. The best cutoff of PLR was 172.10 and the AUC was 0.73, with 70.3% sensitivity and 70.4% specificity (**Figure 2B**). Finally, the predictive accuracy of NLR and PLR in ineligible surgery at first diagnosis in the COPD with lung cancer patients was also analyzed with the ROC curve, the best cutoff of NLR was 3.49, and the AUC was 0.68, with 55.6% sensitivity and 74.3% specificity. The ROC curve for PLR had an AUC of 0.69 with a cutoff value of 172.10, a sensitivity of 63.0%, and s specificity of 73.0% (**Figure 2C**).

### Multivariate analyses of risk factors of COPD with lung cancer

To identify the risk factors related to lung cancer in patients with COPD, univariate and stepwise multivariate logistic regression analyses were performed. In the subgroup analysis, the results showed that age (≥70) (OR: 0.291; 95% CI: 0.179–0.474; *P* < 0.001), current smoker (OR: 1.868; 95% CI: 1.143–3.053; *P* = 0.013), FEV1%pred (OR: 1.049; 95% CI: 1.038–1.061; *P* < 0.001), NLR ≥2.91 (OR: 12.731; 95% CI: 7.493–21.631; *P* < 0.001), PLR ≥156.53 (OR: 4.389; 95% CI: 2.609–7.382; *P* < 0.001), and emphysema (OR: 2.815; 95% CI: 1.560–5.080; *P* = 0.001) were independent risk factors related to the COPD with lung cancer patients (**Table 2A**). Then, we evaluated the risk factors associated with TNM stages IIIB–IV at first diagnosis in the COPD with lung cancer patients. On multivariate analysis, current smoker (OR: 6.583; 95% CI: 2.941–14.736; *P* < 0.001), NLR ≥3.53 (OR: 3.788; 95% CI: 1.811–7.923; *P* < 0.001), and PLR ≥172.10 (OR: 4.775; 95% CI: 2.321–9.823; *P* < 0.001) were independent predictors of TNM stages IIIB–IV at first diagnosis in the COPD with lung cancer patients (**Table 2B**). Finally, we extended our analysis to identify the factors that contributed to ineligible surgery at first diagnosis in the COPD with lung cancer patients. BMI ≥25 (kg/m^2^) (OR: 0.219; 95% CI: 0.061–0.792; *P* = 0.021), current smoker (OR: 4.267; 95% CI: 2.051–8.875; *P* < 0.001), and PLR ≥172.10 (OR: 3.268; 95% CI: 1.531–6.976; *P* = 0.002) were the statistically significant risk factors for ineligible surgery treatment in the COPD with lung cancer patients (**Table 2C**).

## Discussion

In the present study, we investigated the clinical features of COPD-associated lung cancer in a large cohort of COPD patients and identified the value of NLR or PLR that served as an independent predictive factor in COPD with lung cancer. The results indicated that age, smoking status, pulmonary function, serum inflammation biomarkers, and emphysema were associated with COPD coexisting with lung cancer. In terms of median age, the lung cancer group was younger than the COPD group. Airflow limitation assessed by FEV1%pred in COPD was more severe than in lung cancer. The possible reason is that low-dose spiral computed tomography (LDCT) screening was initiated in the year 2010 in China, based on the development of the National Lung Cancer Screening Program (NLST) (34). LDCT was considered as a prevalent screening tool for the early detection of lung cancer in high-risk asymptomatic individuals (35, 36). Another possible reason is that spirometry screening for airflow obstruction in asymptomatic smokers is still controversial (37). Many smokers never experienced lung function decline during the early stage (38). Therefore, based on CT, screening in smoking patients resulted in a higher detection rate of early-stage lung cancer, especially for mild-to-moderate COPD. It has been previously reported that smokers with COPD have a two- to fourfold higher risk of developing lung cancer compared with smokers without COPD (39, 40). It was elucidated that the prevalence of lung cancer was significantly higher in patients with COPD than in the average non-smoking population, reflecting the impact of cigarette smoking on both diseases (41). It has been well acknowledged that cigarette smoking exposure which caused lung barrier dysfunction and inflammation-associated COPD should be considered as an important risk factor for lung cancer (42). In our research, current smoking was an independent risk factor for lung cancer in the COPD group and related to TNM stages IIIB–IV and ineligible surgery treatment. Furthermore, there have been several studies showing that emphysema was an independent poor prognostic factor for tumor recurrence in completely resected NSCLC patients (43). Emphysema severity and centrilobular subtype were associated with a greater risk of lung cancer (44). It was considered that emphysema is an independent predictor of lung cancer diagnosis (45). Other researchers have shown that quantitative emphysema severity of the whole lung and stage III were independent predictors of lung cancer recurrence after adjusting for age, gender, smoking status, and FEV1 (46). Lung adenocarcinomas in the emphysema group have a more aggressive pathologic grade and a higher prevalence of solid lesions (47). Consistent with previous studies, our study showed that emphysema was also independently associated with lung cancer coexisting with COPD.

On the other hand, COPD is characterized as a chronic systemic inflammatory disease, which also has been found to be related to exacerbation (48). Several reports have shown that peripheral blood neutrophils and T lymphocytes were activated and there were increased levels of proinflammatory cytokines in the plasma (49–51). A recent study also demonstrated that increased baseline neutrophil counts were significantly associated with worse OS but not for lymphocyte counts in NSCLC (52). Wong et al. reported positive associations between white blood counts and lung cancer risk among never-smoking women (53). Furthermore, another study found that lymphocyte percentage exhibited a high correlation with the clinical characteristics and metastasis of lung cancer patients (54). In this study, we observed that circulating white blood counts and neutrophil counts were significantly higher in the lung cancer group than in the COPD-only group. It was likely that neutrophils were quickly recruited from the circulation during pulmonary inflammation (55). Thus, we have considered that persistent inflammation could mediate the progression of COPD with lung cancer. Several reports have shown that the levels of NLR or PLR significantly increased in the lung cancer and COPD groups, respectively (56, 57). However, few studies have reported the levels of NLR or PLR in lung cancer with COPD. Our data revealed that NLR and PLR levels were significantly higher in the COPD with lung cancer group than in the COPD group. Then, to further confirm the clinical relevance of inflammation biomarkers, we analyzed the correlation between NLR, PLR, and clinical data from lung cancer patients with COPD. Some studies showed that elevated PLR was associated with shorter OS and poor PFS in NSCLC (58, 59). Another study suggested that elevated PLR might be a predictive factor of poor prognosis for NSCLC patients (60). Yao et al. found that the levels of NLR and PLR were significantly higher among non-survivors compared to survivors of AECOPD, and NLR may be a simple and useful prognostic marker for hospital mortality in patients with AECOPD (61). Elevated NLR could be used as a marker similar to CRP, WBC, and ESR in the determination of increased inflammation in acutely exacerbated COPD and could be beneficial for the early detection of potential acute exacerbations in patients with COPD who have normal levels of traditional markers (62). In the present study, the levels of NLR and PLR had statistical significance among the multiple stratifications in clinical parameters, especially those with a high NLR or PLR were associated with age over 70 years old, current smoking status, and ineligible surgery treatment. The level of PLR or NLR markedly increased with the severity of airflow limitation accessed with GOLD stages 3–4 and advanced progression of lung cancer evaluated with TNM stages IIIB–IV. Additionally, NLR or PLR was positively associated with age, FIB, and D-dimer. We also found a negative association between NLR and FEV1%pred. PLR was negatively associated with BMI and FEV1%pred. The results revealed that PLR or NLR values were inversely associated with the severity of airflow limitation. Also, it was suggested that higher PLR or NLR was positively associated with hypercoagulation status. In our study, the applicable thresholds for NLR and PLR were observed using the ROC curve. Multivariate analysis revealed that NLR ≥2.91 and PLR ≥156.53 were independent risk factors for lung cancer development in COPD patients; the COPD with lung cancer patients with NLR ≥3.53 and PLR ≥172.10 are more likely to be diagnosed in TNM stages IIIB–IV at first diagnosis, while patients with PLR ≥172.10 had fewer surgical opportunities. We believe that NLR or PLR could be part of an important and easily measurable inflammation biomarker system to predict the diagnosis and severity of COPD with lung cancer.

However, this study also had several limitations. Because the pulmonary function tests cannot be properly performed on a number of elderly people in this study, this may lead to the possibility of selection bias in the COPD population. Another limitation is the lack of data on follow-up survival information. We did not evaluate the outcomes of the surgical treatment and did not perform a prognostic analysis due to the high rate of missing follow-up data. Longitudinal studies are needed to evaluate the long-term clinical prognosis of COPD with lung cancer. In addition, the study has a retrospective design, in which a number of patients with severe COPD were unable to undergo lung tissue pathologic diagnosis. Thus, it may have caused bias in the selection of the study group. Moreover, this study could not provide information about the use of inhaled corticosteroids, which is a potential influencing factor for the prevention of lung cancer (63, 64).

## Data availability statement

The raw data supporting the conclusions of this article will be made available by the authors, without undue reservation.

## Ethics statement

The studies involving human participants were reviewed and approved by Ethical Committee of the Clinical Research Ethics Committee of the First Affiliated Hospital of Xiamen University. The patients/participants provided their written informed consent to participate in this study.

## Author contributions

AM and WG drafted the manuscript. GW and YD collected the associated clinical data. HH and LZ contributed to the data statistical analyses. DB and YH prepared the figures and tables. JC and QL wrote the manuscript and conceptualized the framework for this research. All authors read and approved the final manuscript.

## Funding

This work was supported the Xiamen Science and Technology Bureau (3502Z20194004) and Xiamen Science and Health Joint Project of Fujian Natural Science Foundation (2020J011229).

## Acknowledgments

The authors would like to thank all the patients who participated in this study.

## Conflict of interest

The authors declare that the research was conducted in the absence of any commercial or financial relationships that could be construed as a potential conflict of interest.

## Publisher’s note

All claims expressed in this article are solely those of the authors and do not necessarily represent those of their affiliated organizations, or those of the publisher, the editors and the reviewers. Any product that may be evaluated in this article, or claim that may be made by its manufacturer, is not guaranteed or endorsed by the publisher.
