# Supplementary material for: The clinical relevance of neutrophil-to-lymphocyte ratio and platelet-to-lymphocyte ratio in chronic obstructive pulmonary disease with lung cancer
Source: Front Oncol. 2022 Sep 27;12:902955. doi: 10.3389/fonc.2022.902955 (PMC9552820; doi:10.3389/fonc.2022.902955)
Supplement: Supplementary file 1 [file DataSheet_1.docx]

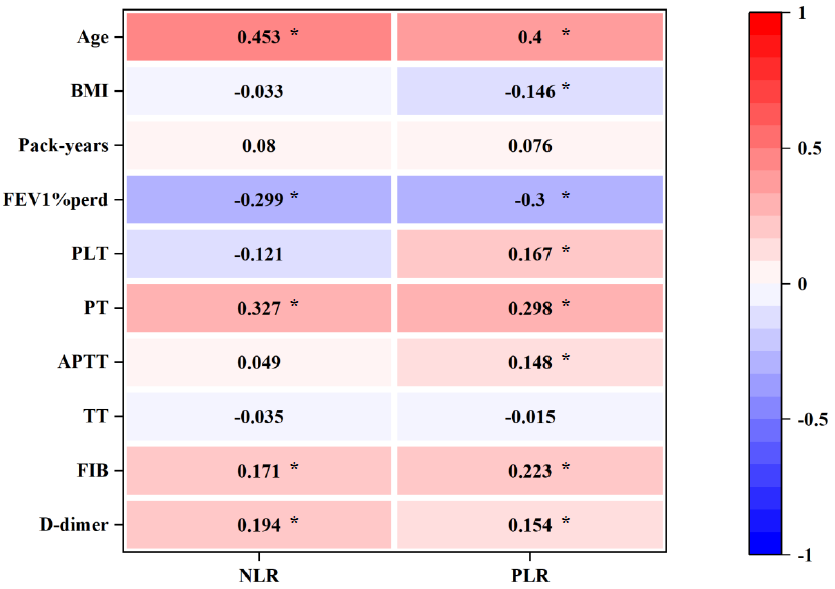


**FIGURE 1** Correlations of NLR and PLR with different parameters in COPD with lung cancer

Abbreviations: PLT, platelet; PT, prothrombin time; APTT, activation partial thrombin time; TT, thrombin time; FIB, fibrinogen. *P-value <0.05.

**TABLE 1 Clinicopathological characteristics of COPD patients with lung cancer**

| **Clinicopathological characteristics** | **COPD with Lung Cancer**  **（n=236）** |
| --- | --- |
| **Histopathology types** |  |
| Adenomatous carcinoma, n (%) | 121(51.3) |
| Squamous cell carcinoma, n (%) | 73(30.9) |
| Aden squamous carcinoma, n (%) | 3(1.3) |
| Large cell lung cancer, n (%) | 8(3.4) |
| Small cell lung cancer, n (%) | 31(13.1) |
| **Locations of pulmonary** |  |
| Left, n (%) | 104(44.1) |
| Left upper lung field, n (%) | 67(28.4) |
| Left lower lung field, n (%) | 37(15.7) |
| Right, n (%) | 132(55.9) |
| Right upper lung field, n (%) | 70(29.4) |
| Right middle lobe of lung field, n (%) | 16(6.8) |
| Right lower lung field, n (%) | 46(19.7) |
| **TNM** |  |
| Ⅰ, n (%) | 60(25.4) |
| Ⅱ, n (%) | 26(11.0) |
| Ⅲ, n (%) | 53(22.5) |
| Ⅵ, n (%) | 97(41.1) |

Abbreviations: TNM, Tumour, Node, Metastasis.

**TABLE 2 The relationship between NLR, PLR levels and clinical characteristics in COPD with lung cancer.**

| **Clinical characteristics** | | **NLR** | **P-value** | **PLR** | **P-value** |
| --- | --- | --- | --- | --- | --- |
| **Age** | <70 | 3.22±0.53 | <0.001 | 163.45±21.66 | <0.001 |
|  | ≥70 | 4.03±0.71 |  | 188.89±22.01 |  |
| **Gender** | Female | 3.63±0.28 | 0.533 | 171.31±22.61 | 0.834 |
|  | Male | 3.51±0.74 |  | 172.80±25.14 |  |
| **BMI** | <25（Kg/M^2^） | 3.50±0.70 | 0.483 | 173.25±24.54 | 0.229 |
|  | ≥25（Kg/M^2^） | 3.63±0.91 |  | 165.46±30.15 |  |
| **Smoking status** | Current smoking | 3.63±0.68 | <0.001 | 166.76±24.09 | 0.036 |
|  | Never/ever smoking | 3.17±0.74 |  | 174.66±25.00 |  |
| **GOLD stage** | 1-2 | 3.39±0.68 | <0.001 | 168.32±24.72 | <0.001 |
|  | 3-4 | 3.93±0.69 |  | 188.28±19.03 |  |
| **TNM stage** | Ⅰ-ⅢA | 3.18±0.58 | <0.001 | 163.45±22.82 | <0.001 |
|  | ⅢB-Ⅳ | 3.79±0.70 |  | 180.54±24.07 |  |
| **Treatment** | Eligible-surgery | 3.17±0.62 | <0.001 | 163.02±23.95 | <0.001 |
|  | Ineligible-surgery | 3.67±0.71 |  | 177.15±24.22 |  |

Abbreviations: BMI, body mass index; GLOD, Global Initiative for Chronic Obstructive Lung Disease.
